# Some Points of Interest in the Surgical Department of the Municipal Hospital at Frankfort

**Published:** 1903-05-09

**Authors:** 


### SOME POINTS OF INTEREST IN THE SURGICAL DEPARTMENT OF THE MUNICIPAL HOSPITAL AT FRANKFORT.

BY A CORRESPONDENT.

(*Continued from page* 67.)

### The Supply of Normal Saline Solution.

The next splendid arrangement is that for the supply of the normal saline solution. The apparatus in which the solution is mixed, sterilised, and stored, stands in a small room above the body of the operating theatre, and holds about 250 litres. After having been sterilised and cooled by cold coils running through the boiler, the normal saline solution is kept at a constant temperature of 40° C. by means of a gas and mercury regulator. The ceiling of the operating theatre is pierced, and a large indiarubber tube brings the solution straight from the apparatus to the operating table. This tube is sterilised by driving steam under pressure through it, and, in addition, the lower part of the tube is boiled in the theatre. The instrument case stands on four high legs let into the floor, and is in a central position, accessible from all sides. Sterilised water is supplied by a pipe which receives steam at the engine-house, and this is condensed on the way. Another tap admits steam into the theatre itself, and this is turned on every morning before operations for 20 minutes, so as to destroy every vestige of dust. A hose for washing down the walls and ceiling is used after operations. It may be of interest to our readers to know the method of disinfecting hands and arms in this theatre: The sleeves are rolled up above the elbow, and a Billroth battiste—thin oilcloth—overall put on. Then the water is turned on, the sand clock started, and continual scrubbing of arms and hands with liquid potash soap constitutes the first part of the cleansing process. When this has been done for five minutes, a nurse whose hands have been subjected to all the stages of disinfecting, puts on a pair of sterile gloves, and helps the doctors and fellow nurses into the sterilised overalls. She then takes off the gloves, and goes through the following two stages of disinfecting with the rest of the party. The hands and arms are next immersed into basins with absolute alcohol for three minutes, and then into lotio hydrarg: perchlor: 1 in 1,000 for two minutes. This completes the process, and the theatre sister assured me that cultivations, taken from their disinfected skins, almost invariably remained sterile. Gloves are scarcely ever used by surgeon or assistants. The anæsthetic room adjoins the theatre, and contains, in addition to the ordinary supply, a well stocked equipment for counteracting shock, including electric brushes for the heart, etc. For administering anæsthetics a chloroform and oxygen apparatus is very much in favour at present. Altogether chloroform and ether are used almost exclusively at Frankfort as general anæsthetics, the English A.C.E mixture is scarcely known, and gas is not popular. On the other hand eucaine and other local anæsthetics are employed largely. The sterilising room, opposite the theatre, is furnished with a row of sterilisers, for which steam under pressure is used, and which are easily worked by a nurse. In addition there is a hot-air steriliser for the dishes and basins. The original drum-shaped Schimmelbusch boxes are still the fashion for keeping dressings, whereas towels, overalls, and such articles are enveloped in thick calico wraps. The dressing materials are the ordinary absorbent wool and gauze, and another equally absorbent gauze of a more crêpe-like consistency—krüllgaze. Swabs are made from this latter material, and the iodoform gauze is prepared by the nurse herself from the ordinary absorbent gauze. A very complete $x$-ray room and a room where bandages are rolled and dressings are got ready for sterilising, complete the operating-theatre department.

### The Septic Block.

When we leave the surgical building proper we come to the septic block, into which all cases which are not absolutely clean to start with are admitted. This department contains wards, dressing-rooms, etc., fitted exactly like those of the aseptic block, and, in addition, a number of small wards and single rooms for isolation cases. The most striking feature of this wing is the division for permanent baths. These are furnished with extra large white porcelain baths, into which fits a very comfortable stretcher on legs. The water-supply comes from a room above, where it is regulated to 40° C., and enters the top of the bath in a gentle, continuous stream, while water leaves the bath at the lower end. The patients are covered over and look most comfortable, with reading-desks going across the width of the bath. The stretchers are lowered into and lifted out of the baths by means of a crane, which can be easily managed by one nurse, who turns a handle on the wall of the bath-room. It may interest our readers to hear that a scheme has recently been sanctioned by the Government, and also approved of by the town fathers, to establish, in connection with this model hospital, a Post-Graduate Medical Teaching School. So far, no medical school is attached to the hospital. The beds will then be increased to 1,200, various new blocks for special diseases—women, throat, ears, eyes, etc.—will be erected; an institute for serum-therapeutics, pathological anatomy, hygiene, etc., will be included in the so-called "Academy of Medicine," and so this new institution—the first of its kind in Germany and with an assured stock of teachers like Ehrlich, Weigert, Noorden, Rehn, who are already in charge of the most important departments—bids fair to be a great success, and at the same time, we hope, a boon to suffering humanity.

